# Self-amputation: Case report

**DOI:** 10.1192/j.eurpsy.2021.966

**Published:** 2021-08-13

**Authors:** I. Cuevas Iñiguez, M.D.C. Molina Lietor

**Affiliations:** Psiquiatría, Hospital Universitario Príncipe de Asturias, Alcalá de Henares, Spain

**Keywords:** Self-amputation, differential diagnosis, case report

## Abstract

**Introduction:**

Self-amputation, the most severe form of self-mutilation, is unusual. In most cases, self-mutilation is related to psychiatric disorders, mainly psychotic spectrum disorders and substance abuse.

**Objectives:**

This case report aims to describe a case of unusual self-amputation in a man with a psychiatric history.

**Methods:**

Case report and literature review.

**Results:**

A 35 years old man patient, divorced, unemployed, with 15 years of treatment history for anxiety and low mood. The patient reported history of childhood trauma. He was inpatient (2019) after a suicide attempt. The psychiatrist who was treating him did not give a diagnosis (referral diagnosis). The patient mentioned several times that he desired feet amputation, without planification, in context of high anxiety. He was distressed by the shape and noise of his ankles. The patient was not diagnosed with genuine hallucinations or delusions. Four months after his divorce he amputated his feet with an electric saw. He denied any intention to commit suicide by committing this act. He admitted that he wanted to get rid of discomfort. Despite this drastic action, his mood did not improve.

**Conclusions:**

Self-amputation is not a common condition. Although some cases of self-amputation have been reported, this case illustrates not only the difficulty of making a differential diagnosis (psychosis, dissociation, trauma, dysmorphophobia, body identity integrity disorder…) but also the challenge of a multidisciplinary approach in the treatment of patients with self-amputations.

